# Spontaneous point mutations in the capsule synthesis locus leading to structural and functional changes of the capsule in serogroup A meningococcal populations

**DOI:** 10.1080/21505594.2018.1467710

**Published:** 2018-08-01

**Authors:** Emma Ispasanie, Francesca Micoli, Araceli Lamelas, Dominique Keller, Francesco Berti, Riccardo De Riccio, Roberta Di Benedettoi, Simona Rondini, Gerd Pluschke

**Affiliations:** a Swiss Tropical and Public Health Institute, Molecular Immunology Unit, Basel, Switzerland; b University of Basel, Basel, Switzerland; c GSK Vaccines Institute for Global Health, Siena, Siena, Italy; d Red de Estudios Moleculares Avanzados, Instituto de Ecologia, A.C., Veracruz, México; e GSK, Siena, Italy

**Keywords:** *Neisseria meningitidis*, capsule synthesis locus, genomic diversity, herd immunity, virulence

## Abstract

Whole genome sequencing analysis of 100 *Neisseria meningitidis* serogroup A isolates has revealed that the *csaABCD-ctrABCD-ctrEF* capsule polysaccharide synthesis locus represents a spontaneous point mutation hotspot. Structural and functional properties of the capsule of 11 carriage and two disease isolates with non-synonymous point mutations or stop codons in capsule synthesis genes were analyzed for their capsular polysaccharide expression, recognition by antibodies and sensitivity to bactericidal killing. Eight of eleven carriage isolates presenting capsule locus mutations expressed no or reduced amounts of capsule. One isolate with a stop codon in the O-acetyltransferase gene expressed non-O-acetylated polysaccharide, and was not recognized by anti-capsule antibodies. Capsule and O-acetylation deficient mutants were resistant to complement deposition and killing mediated by anti-capsular antibodies, but not by anti-lipopolysaccharide antibodies. Two capsule polymerase mutants, one carriage and one case isolate, showed capsule over-expression and increased resistance against bactericidal activity of both capsule- and lipopolysaccharide-specific antibodies. Meningococci have developed multiple strategies for changing capsule expression and structure, which is relevant both for colonization and virulence. Here we show that point mutations in the capsule synthesis genes substantially contribute to the repertoire of genetic mechanisms in natural populations leading to variability in capsule expression.

## Introduction

*Neisseria meningitidis* is an obligate human pathogen that colonizes the human nasopharyngeal mucosa and occasionally invades the bloodstream, causing severe invasive disease such as septicemia or meningitis [1]. Countries of the African ‘meningitis belt’ have for over 100 years experienced large meningococcal meningitis epidemics [2–4]. Multilocus sequence typing (MLST) of carriage and disease isolates has shown that between the late 1980s and 2010, epidemic waves of colonization and disease were mainly associated with hypervirulent serogroup A clones with the sequence types (ST) 5, 7, and 2859 [5–8]. The ST-5 clone spread in the meningitis belt after an epidemic in Mecca in 1987. The ST-7 and the ST-2859 clone were found in Africa for the first time in 1995 and 2003, respectively [9–11]. Comparative whole genome sequencing analyzes showed that the ST-2859 lineage evaded herd immunity elicited by their ST-7 ancestors primarily through rapid homologous replacement of a set of genomic loci affecting non-capsular cell surface components [12].

The closely related A clones, namely, ST-5, 7 and 2859 followed each other in different geographical settings, each colonizing the local population for a few years before being replaced [13–15]. Due to horizontal gene transfer and point mutations, clonal meningococcal populations are, even in the course of an epidemic, not absolutely identical. Analysis of ST-7 and ST-2859 serogroup A isolates isolated between 2001 and 2009 in the framework of longitudinal meningococcal colonization and disease surveys in Ghana and Burkina Faso identified the *pilT-pilU* locus, which is implicated in the regulation of type IV pilus expression, and the *csaABCD-ctrABCD-ctrEF* capsule synthesis locus (cps) as point mutation hot spot regions [12].

The capsular genes are clustered within the *cps* chromosomal locus and divided into six regions, designated A-D, D’ and E. Genes in region A (*csaABCD*) encode for polysaccharide biosynthesis and polymerization enzymes [16]. Proteins encoded by genes in region B (*ctrE and ctrF*) and C (*ctrABCD*) are involved in translocation of the polysaccharide from the cytoplasm to the cell surface. Region D encoded proteins are required for lipopolysaccharide (LPS) synthesis. Genes in region D’ encode methyltransferases and region D genes which are duplicated and truncated (*galE*). The function of region E is unknown. The serogroup A polysaccharide consists of repeating units of (α1-6)-linked *N-*acetyl-mannosamine-1-phosphate synthesized by enzymes encoded by genes designated *mynA-D* or *csaA-D* [16–18]. The repeating units are O-acetylated mainly at position C3 and sometimes at position C4 [17].

Meningococci have developed a range of genetic mechanisms to modify expression and structure of their capsule [19]. These include phase variation by slipped-strand miss-pairing, transcriptional regulation, post-transcriptional regulation via RNA thermosensors, insertion element (IS) associated on-off switching and ‘capsule switching’ by horizontal gene transfer. Among the 100 clonally related serogroup A isolates analyzed by comparative whole genome sequencing, Lamelas et al. [12] identified 13 isolates with non-synonymous single nucleotide polymorphisms (SNPs) or stop codons in the capsule synthesis locus. In contrast, none of the 100 sequenced isolates carried a synonymous point mutation in this locus, speaking for selection of variants. Here we describe the phenotypic, structural and functional effects caused by the non-synonymous point mutations and stop codons in the *cps* locus.

## Materials and methods

### Neisseria meningitidis isolates

The serogroup A *N. meningitidis* isolates investigated in this study (Table 1) have been collected in the framework of longitudinal meningococcal colonization and disease studies in the Kassena-Nankana District (KND) and the neighboring Bolgatanga district (BA) hospital of Ghana and in the Nouna Health District (NHD) of Burkina Faso. Case isolates were obtained from the cerebrospinal fluid of meningitis patients and carriage isolates from throat swabs. For the longitudinal meningococcal colonization studies with a sample size of about 300 participants, residential compounds in the KND and the NHD were selected and throat swabs were taken twice annually from all consenting members of the selected compounds present at the time of the visit [15,20,21]. Isolation and characterization of bacterial isolates [15,20,21], and the comparative whole genome analysis of these isolates [12] have been described previously. Ethical clearance was obtained from the relevant institutional review boards and the Ministries of Health. Informed consent for use of the samples was obtained from all study participants.

**Table 1.** Serogroup A *Neisseria meningitidis* capsule locus mutants (n = 13) and reference isolates (n = 3) and properties of their capsular polysaccharide.

Isolate (ST)	Accession Number	Origin	Mutated gene	Base change	Amino acid change	Affected function	Anti-A capsule mAb staining*	Amount of capsule (µg/mL/OD)	O-acetylation (%)	Bactericidal activity of anti-ACWY serum (Mean titre)	Bactericidal activity of anti-LPS mAb (Mean titre)
1976 (7)	ERS041005	2004, KND, pharynx	*ctrE* non-syn.	1529^T->C^	L510P	translocation	negative	<1.5	na	negative	positive (65,020)
2389 (2859)	ERS041030	2006, NHD, pharynx	*csaD* stop	758^G->A^	W253X	transport/cross-linking	negative	<1.5	na	negative	positive (29,440)
1921 (7)	ERS040999	2004, KND, pharynx	*csaB* stop	915^G->A^	W305X	polymerase	negative	<1.5	na	negative	positive (1.2x10^7)
2206 (2859)	ERS041015	2006, NHD, pharynx	*csaB* non-syn	1157^G->A^	G386D	polymerase	negative	<1.5	na	negative	positive (20,914)
2856 (2859)	ERS041053	2008, NHD, pharynx	*csaB* non-syn	427^C->T^	P143S	polymerase	normal	6.7	not done	positive (9093)	positive (541,935)
2857 (2859)	ERS041054	2008, NHD, pharynx	*csaB* non-syn	427^C->T^	P143S	polymerase	normal	6.8	40	positive (19,397)	positive (1.5x10^6)
2717 (2859)	ERS041044	2007, NHD, pharynx	*csaA* non-syn	941^A->C^	E314A	epimerase	reduced	8.5	40	negative	positive (131,164)
2617 (2859)	ERS041041	2006, NHD, pharynx	*csaA* non-syn	417^C->G^	S139R	epimerase	normal	22.7	55	positive (97,813)	positive (982,603)
1471 (7)	ERS040970	2002, KND, pharynx	*csaB* non-syn	176^C->A^	S59Y	polymerase	normal	41.7	60	positive (3319)	positive (595,269)
2025 (7)	ERS041008	2005, KND, pharynx	*csaB* non-syn	244^A->G^	I82V	polymerase	above normal	71.2	not done	reduced (49)	reduced (25)
2008 (7)	ERS041006	2005, KND, CSF	*csaB* non-syn	244^A->G^	I82V	polymerase	above normal	61.4	55	reduced (80)	reduced (25)
1666 (7)	ERS040979	2003, BA, pharynx	*csaC* stop	490^C->T^	Q164X	O-acetyl transferase	negative	39.7	no OAc	negative	positive (4.5x10^6)
1573 (7)	ERS040977	2003, KND, CSF	*ctrD* non-syn	185^T->C^	I62T	export	normal	42.2	50	positive (4064)	positive (412,851)
1446 (7)	ERS040969	2002, KND, pharynx	reference isolate	none	none	none	normal	34.9	not done	positive (278,672)	positive (749,690)
2187 (2859)	ERS041012	2006, NHD, CSF	reference isolate	none	none	none	normal	51.8	60	positive (8281)	positive (511,796)
2602 (2859)	ERS041039	2007, NHD, CSF	reference isolate	none	none	none	normal	58.1	50	positive (15,840)	positive (65,020)

The amount of capsular polysaccharide was quantified by HPAEC-PAD. Nuclear Magnetic Resonance (NMR) analysis was used to determine the O-acetylation of the cell bound capsule. Abbreviations: non-syn = non-synonymous; na = not analyzable due to lack of polysaccharide; OD = optical density; mAb = monoclonal antibody; Abs = antibodies; CSF = cerebrospinal fluid; KND = Kassena-Nankana District, Ghana; BA = Bolgatanga district, Ghana; NHD = Nouna Health District, Burkina Faso.

*Flow cytometry was used to determine the capsule expression of the mutant isolates relative to the reference isolates.

### Monoclonal antibodies and antisera

Polyclonal antisera reactive with the serogroup A capsule were obtained from mice immunized with the Menveo A, C, W, Y capsule polysaccharide conjugate vaccine (anti-ACWY capsule antiserum). The *N. meningitidis* LPS specific monoclonal antibody (mAb) EI9.2 of the IgG subclass IgG2a was produced in-house (anti-LPS mAb), and the serogroup A capsule specific mAb 95/674 (anti-A capsule mAb) [22] was obtained from the National Institute for Biological Standards and Control (Potters Bar, Hertfordshire, UK).

### Analysis of capsule expression by flow cytometry

Isolates were cultured at 37°C, 5% CO_2_ to an OD_600_ of 0.7 in Mueller-Hinton broth (MH; Becton Dickinson AG; Allschwil, Switzerland), washed, inactivated with 4% (v/v) formalin and re-suspended in Dulbecco’s phosphate buffered saline containing 0.5% (w/v) bovine serum albumin (DPBS-BSA, Sigma-Aldrich, St. Louis, MO, USA). Cell suspensions were incubated with 10 μg/mL anti-A capsule mAb 95/674 or with a 1:50 dilution of the polyclonal anti-ACWY capsule antiserum for 1 hour at room temperature. Alexa Fluor 488-conjugated goat anti-mouse IgG (heavy and light chain) specific antibodies (Invitrogen, Carlsbad, CA, USA) were used as secondary antibody at a 1:200 dilution. Cells re-suspended in FACS flow buffer (Becton Dickinson AG) were analyzed using a FACS Calibur (Becton Dickinson AG) FL2-H channel and the software CellQuest Pro version 5.2.1 (Becton Dickinson AG). For all experiments, the cells were gated at 30,000 events. Results were reproduced in two independent experiments.

### Flow cytometric analysis of C3b and C4b deposition

For C3b and C4b deposition experiments, 100 μL of formalin inactivated bacterial cells in DPBS-BSA were incubated with polyclonal anti-ACWY capsule antiserum at a 1:50 dilution or with 4 μg/mL anti-LPS mAb for 1 hour at room temperature (C3b) or at 4°C (C4b). Subsequently, 2% human serum from a healthy individual with no detectable bactericidal activity against the test isolates was added as complement source. The serum was depleted of IgG by passing it over a HiTrap rProtein G FF column (GE Healthcare Life Sciences, Little Chalfont, UK) and filter sterilized. Removal of IgG was verified by enzyme-linked immunosorbent assay (ELISA). After 15 minutes of incubation at 4°C, 100 μL of FITC conjugated rabbit polyclonal anti-C3c antibodies (ab4212, diluted 1:2,000; Abcam, Cambridge, UK) or anti-C4c + C4b antibodies (ab4216, diluted 1:3,000; Abcam) were added for 1 hour at 4°C. Surface staining was analyzed using the FACS Calibur FL1-H channel.

### Bactericidal assay

The assay was performed as previously described [23]. Reaction mixtures contained approximately 400 colony-forming units (CFU), 20% human serum with no detectable bactericidal activity against the test isolates as complement source and serial dilutions of the anti-ACWY capsule antiserum or the anti-LPS mAb in serial 4-fold dilutions (stock 1 mg/mL). Bactericidal titers were defined as the dilution of serum or mAb resulting in 50% decrease of CFU/mL after 60 minutes incubation at 37°C compared to the CFU/mL in the control reactions at time 0 [24,25]. Antibody titers were log10 transformed, and bactericidal titers <10 were assigned the value 5. Data analysis was carried out using GraphPad Prism version 7.0b software.

### Characterization of capsular polysaccharide produced by the different serogroup A isolates

Capsular polysaccharide identity and O-acetylation levels were analyzed by proton high-resolution magic angle spinning (HR–MAS) nuclear magnetic resonance (NMR). Data were collected on entire bacteria in heterogeneous phase to verify capsular polysaccharide presence and to determine the O-acetylation level. Bacteria were grown overnight in 50 mL of MH medium up to an OD of 4–5. Cultures were treated with p-formaldehyde (final concentration 0.1% w/v) to inactivate the bacteria. Samples were pelleted by centrifugation (4500 g for 2 min) and washed three times with 10 mM potassium phosphate buffer in deuterated water. Each compact pellet was transferred into a Kel-F disposable insert for a 50 µL volume and then spun in a 4-mm MAS ZrO_2_ rotor (Bruker, Billerica, MA, USA). Proton HR–MAS NMR experiments were recorded by a Bruker Avance III 400-MHz spectrometer, using a Bruker 4-mm HR–MAS probe. Samples were spun at 4.5 kHz and recorded at 25°C. Proton spectra were acquired with diffusion filter pulse sequence to remove sharp lines arising from low-molecular-mass species in solution. Spectra were collected with 32 k data points over a 10-ppm spectral width. The transmitter was set at the HDO frequency, which was also used as reference signal (4.79 ppm). The TopSpin 2.1 software package (Bruker) was used for data acquisition and processing of all spectra was done with MestReNova 10.0.

The amount of capsular polysaccharide was quantified by high-performance anion-exchange chromatography with pulsed amperometric detection (HPAEC-PAD). Samples were desalted on PD-10 desalting columns (GE Healthcare Life Sciences) and treated by acid hydrolysis with trifluoroacetic acid at a final concentration of 2 M in order to release the capsule repeating units without their degradation. Samples were heated at 100°C for 2 hours, then chilled at 2–8°C for about 30 minutes, 700 μL 2 M NaOH were added and samples filtered with 0.45 µm Acrodisc® (PALL, Port Washington, NY, USA) filters before analysis. A pure preparation of serogroup A polysaccharide obtained from GSK Manufacturing (Siena, Italy) was used for building a calibration curve in a range of 0.5–8 µg/mL. The HPAEC-PAD was performed with a Dionex ICS3000 equipped with a CarboPac PA1 column (4 x 250 mm; Dionex, Sunnyvale, CA, USA) coupled with a PA1 guard column (4 x 50 mm; Dionex). Samples were run with a flow rate of 1 mL/min, using a gradient in 10 minutes from 100 mM to 500 mM sodium acetate in 100 mM NaOH. The effluent was monitored using an electrochemical detector in the pulse amperometric mode with a gold working electrode and an Ag/AgCl reference electrode. A quadruple-potential waveform for carbohydrates was applied. The resulting chromatographic data were processed using the Chromeleon software 6.8.

## Results

### Capsular polysaccharide expression in spontaneous capsule synthesis gene mutants

**Figure 1.**
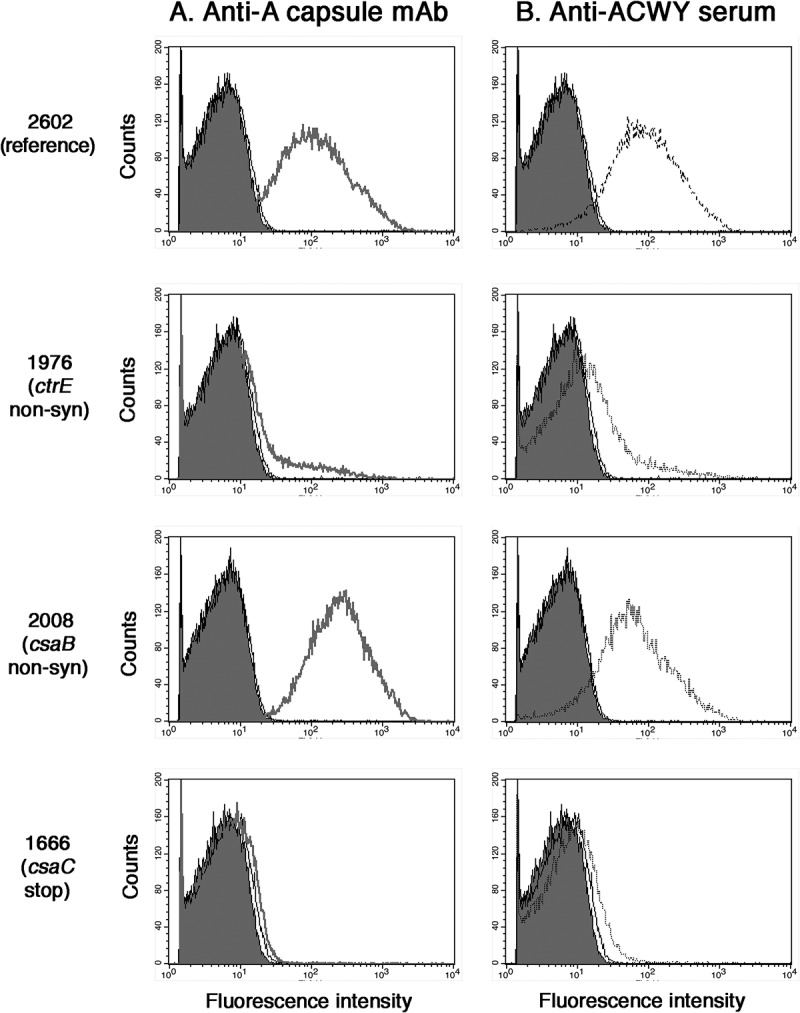
Flow cytometric analysis of the binding of the anti-A capsule specific mAb 95/674 (A) and of mouse anti-ACWY capsule antiserum to meningococcal cells (B). The fluorescence intensity of fixed bacterial cells was determined (counts). Isolate 2602: reference isolate; isolate 1976: no capsule expression in the mutant carrying a non-synonymous SNP in the *ctrE* gene responsible for translocation of the polysaccharide; isolate 2008: increased capsule expression in the mutant carrying a non-synonymous SNP in the *csaB* gene that codes for a capsule polymerase; isolate 1666: lack of binding of antibodies to the mutant carrying a stop mutation in the *csaC* gene that encodes an O-acetyltransferase.

Our whole genome sequencing analysis of 100 clonally related *Neisseria meningitidis* serogroup A ST-7 (n = 50) and ST-2859 (n = 50) case and carriage isolates revealed that the *csaABCD-ctrABCD-ctrEF* capsule polysaccharide synthesis locus represents a spontaneous point mutation hotspot [12]. Thirteen of the 100 analyzed isolates carried spontaneous non-synonymous point mutations or stop codons in the *csaABCD-ctrABCD-ctrEF* capsule polysaccharide synthesis locus. Of these 13 mutants, 11 were carriage isolates and two (1573 and 2008) invasive case isolates (Table 1). Flow cytometric analyzes were firstly performed on five ST-7 and six ST-2859 randomly selected isolates belonging to the 87 of 100 isolates with no point mutations in the capsule synthesis locus [12]. These showed only minor variations in staining intensity with either the anti-A capsule specific mAb 95/674 or an anti-ACWY capsule antiserum (Supplementary Figure 1). For further analysis, three isolates (1446, 2187 and 2602) harbouring wild type *csaABCD-ctrABCD-ctrEF* genes were used as references. Capsule expression of mutants and reference isolates were analyzed both by HPAEC-PAD (Table 1) and by flow cytometry with anti-serogroup A capsule antibodies (Figure 1). The reference isolates expressed between 34.9 and 58.1 µg/mL/OD capsule (Table 1) and there was a good correlation between the measured amounts of O-acetylated capsule polysaccharide and the fluorescence intensity (shown for selected isolates in Figure 1).

The mutant isolate 1976 harbouring a non-synonymous SNP in the *ctrE* gene, which encodes a protein required for translocation and surface expression of the lipidated capsular polysaccharide [26], showed no staining with the capsule-specific monoclonal or polyclonal antibodies (Figure 1). It was also negative in the HPAEC-PAD analysis (Table 1), confirming lack of polysaccharide expression. Equally negative in both assays (Table 1) was isolate 2389 with a stop codon in the *csaD* gene encoding a protein predicted to be involved in either capsule transport or in cross-linking of the capsule to the meningococcal cell surface [16]. In contrast, isolate 1573 harbouring a non-synonymous mutation in the *ctrD* gene, encoding an ATP binding protein involved in capsule transport [16], showed no phenotypic changes and a normal amount of capsule (42.2 µg/mL/OD; Table 1). Isolate 1573 is one of the two case isolates included in the analysis.

In isolates 2717 and 2617 harboring non-synonymous point mutations in the *csaA* gene, which encodes an UDP-*N*-acetyl-D-glucosamine-2-epimerase converting UDP-GlcNAc into UDP-*N*-acetyl-D-mannosamine [27], the amount of capsule was reduced to 8.5 and 22.7 µg/mL/OD, respectively (Table 1).

**Figure 2.**
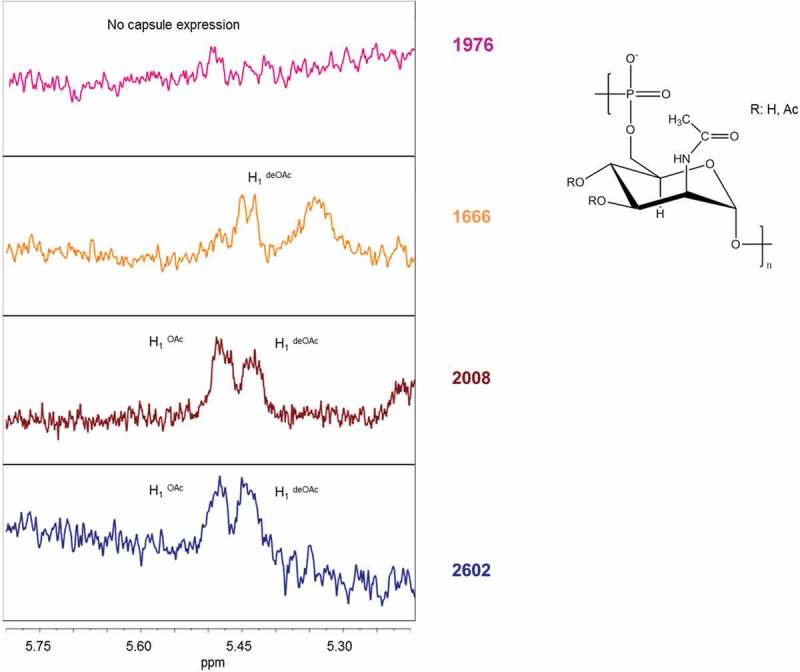
HR MAS-NMR spectra (from 5.2 to 5.8 ppm characteristic for H_1_ signals of O-acetylated (OAc) and not O-acetylated (deOAC) sugar units indicated in the structure of the serogroup A polysaccharide repeating unit of three selected isolates compared to the reference isolate 2602. No capsule expression in isolate 1976 carrying a non-synonymous SNP in the *ctrE* gene responsible for translocation of the polysaccharide; similar O-acetylation level with respect to the reference in isolate 2008 carrying a non-synonymous SNP in the *csaB* gene that codes for a capsule polymerase; no O-acetylation in isolate 1666 carrying a stop mutation in the *csaC* gene that encodes an O-acetyltransferase.

A range of different effects were observed for mutations in the *csaB* gene, encoding a capsule polymerase linking ManNAc-phosphate monomers together [27]. No capsule production was found in isolate 1921 with a stop codon in the *csaB* gene and in isolate 2206, one of the six mutants with a non-synonymous *csaB* point mutation causing a G386D amino acid exchange (Figure 2; Table 1). Reduced capsule expression (<10 µg/mL/OD) was found in two of the other five *csaB* mutants (isolates 2856 and 2857; Table 1) carrying the same mutation causing a P143S amino acid exchange (Table 1). In contrast, an increased capsule expression of 61.4 and 71.2 µg/mL/OD was found in two other non-synonymous *csaB* mutant isolates 2008 and 2025, respectively (shown for the case isolate 2008 in Figure 1). These two isolates were also carrying identical mutations causing an I82V amino acid exchange (Table 1). Only one *csaB* mutant (1471) with an amino acid exchange (S59Y) closer to the N-terminus did not show a change in polysaccharide expression level compared to the reference isolates (Table 1).

Moreover, the O-acetylation levels of the cell associated polysaccharide were determined by HR-MAS NMR (Table 1, Figure 2). The monitoring of the anomeric proton at carbon 1 of the ManNAc-phosphate residue constituting the repeating unit of the serogroup A capsular polysaccharide provided a tool for evaluating levels of O-acetylation [28]. Two separate peaks, one of the O-acetylated form at lower-field signal (5.40 ppm) and the other of the non-O-acetylated form at upper-field signal (5.35 ppm) were resolved (Figure 2). Isolate 1666, with a stop codon in the *csaC* gene encoding an O-acetyltransferase with specificity for the O-3 and O-4 positions in ManNAc [17], expressed normal amounts of capsular material (Table 1), but no O-acetylation was detected (Figure 2). Neither the monoclonal nor the polyclonal capsular polysaccharide antiserum was able to bind to this non-O-acetylated polysaccharide (Figure 1). All other isolates analyzed showed O-acetylation levels between 40 and 60% (Table 1).

### Sensitivity of capsule mutants to antibody mediated bactericidal activity

**Figure 3.**
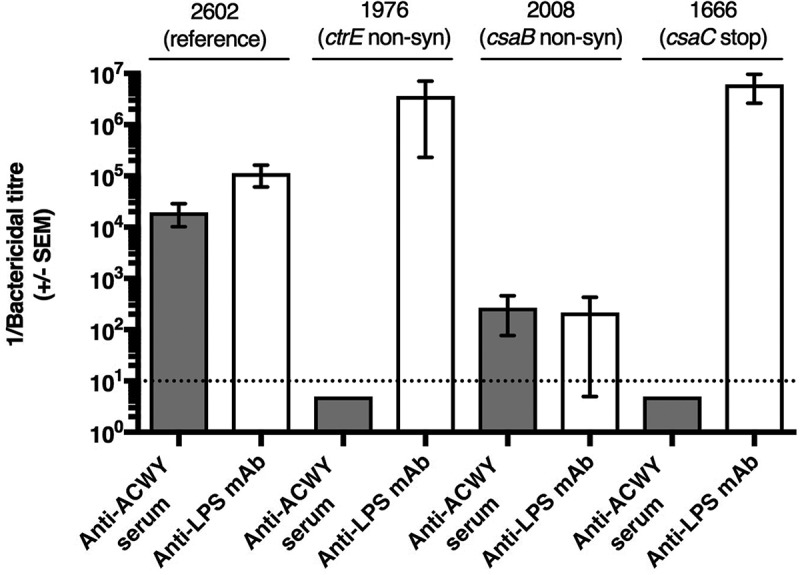
Effect of point mutations in the capsule synthesis locus on bactericidal activity mediated by anti-ACWY capsule antiserum and anti-LPS mAb. Typical results are shown for selected reference and mutant isolates. Reference isolate 2602: sensitive against both anti-ACWY capsule antiserum and anti-LPS mAb; isolate 1976 lacking capsule due to a non-synonymous SNP in the *ctrE* gene: lack of activity of anti-ACWY capsule antiserum; isolate 2008 expressing more polysaccharide than the reference isolate as a result of a non-synonymous mutation in the *csaB* gene: generally reduced bactericidal effect irrespective of the specificity of the antibodies; isolate 1666 expressing non-O-acetylated capsular polysaccharide: lack of activity of anti-ACWY capsule antiserum. Error bars represent standard error of the mean titer of triplicate measurements in three independent experiments. The bottom dotted line indicates the lowest serum dilution (1:10) and highest mAb concentration (100 µg/mL) tested.

The monoclonal anti-A capsule specific mAb 95/674 showed no bactericidal activity, therefore, bactericidal assays were performed with the mouse anti-ACWY capsule antiserum and with the anti-LPS mAb EI9.2. Both were bactericidal for the wild-type control isolates. Due to lack of the target antigen, the polyclonal anti-ACWY capsule antiserum elicited no bactericidal effect against the four mutants deficient in capsule production (isolates 1976, 2206, 2389, 1921) (shown for isolate 1976 in Figure 3) and the *csaA* mutant 2717 with strongly reduced capsule production (Table 1). Likewise, no bactericidal activity was observed with the anti-ACWY capsule antiserum against the O-acetylation deficient *csaC* mutant isolate (1666; Figure 3). In the case of the two isolates with non-synonymous mutations in the *csaB* gene (2025 and 2008) that increased the expression of capsular polysaccharide (Table 1), the bactericidal activity elicited by the polyclonal anti-ACWY capsule antiserum was strongly reduced (shown for isolate 2008 in Figure 3). In contrast to all other isolates, these two over-expressing mutants additionally showed increased resistance to the bactericidal activity mediated by the anti-LPS mAb (Figure 3).

**Figure 4.**
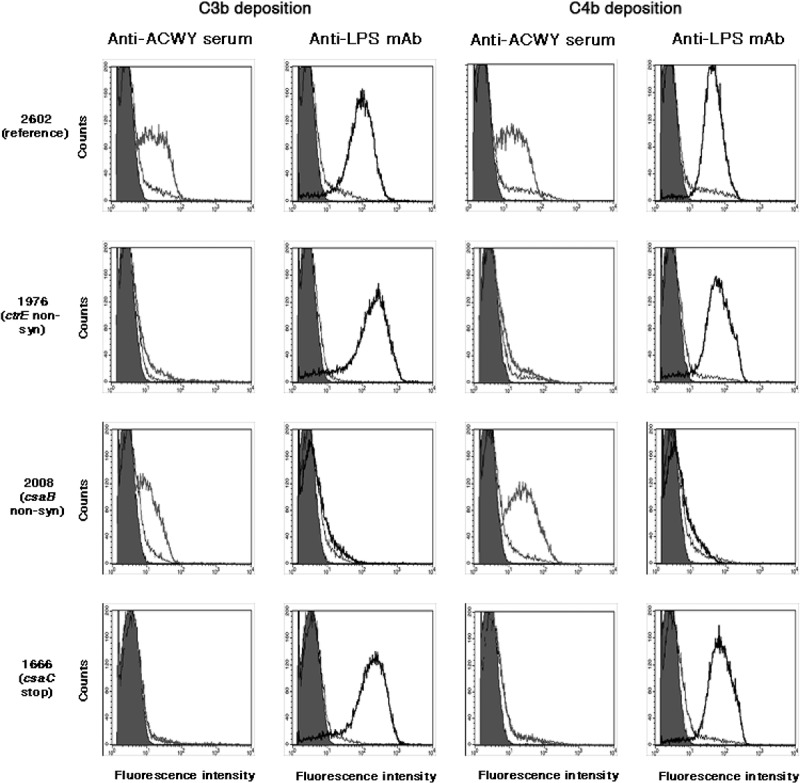
Flow cytometric analysis of the deposition of human C3b and C4b on fixed bacterial cells. Anti-ACWY capsule antiserum (diluted 1:50) or anti-LPS mAb EI9.2 (4 µg/mL) was used together with 5% IgG depleted human serum to investigate the effect of the mutations on antibody mediated C3b and C4b deposition. Typical results are shown for one of the reference isolates and for selected mutant isolates. The fluorescence intensity of fixed bacterial cells was determined (counts). Grey filled area: cells only incubated with secondary antibody; black thin line: negative control with 4 µg/mL of an unrelated IgG1 mAb; grey thick line: anti-ACWY capsule antiserum; black thick line: anti-LPS mAb. Reference isolate 2602: C3 and C4 deposition is caused both by anti-capsule and anti-LPS antibodies; isolate 1976 lacking capsule due to a non-synonymous SNP in the *ctrE* gene: no C3 and C4 deposition with anti-capsule antibodies; isolate 2008 expressing more polysaccharide than the reference isolates as a result of a non-synonymous mutation in the *csaB* gene: C3 and C4 deposition by anti-LPS mAb is strongly reduced; isolate 1666 expressing non-O-acetylated capsular polysaccharide: no C3 and C4 deposition caused by polyclonal anti-ACWY capsule antiserum.

The anti-capsular antibodies were not able to cause C3b and C4b deposition on the surface of isolates lacking capsule (1976, 2389, 1921 and 2206) or having a strongly reduced capsule expression (2717), as shown for isolate 1976 in Figure 4. This correlates well with the observed lack of complement-mediated killing (Figure 3). Likewise, there was no C3b and C4b deposition (Figure 4), no bactericidal activity (Figure 3) and no binding of the anti-ACWY capsule antiserum (Figure 1) with the isolate 1666 deficient in O-acetylation. In contrast, the anti-LPS mAb caused C3b and C4b deposition in these isolates (Figure 4). In the case of the two *csaB* capsule over-expressing mutants 2025 and 2008, C3b and C4b deposition caused by the anti-LPS mAb was strongly reduced (shown for the case isolate 2008 in Figure 4). Anti-ACWY capsule antiserum bound strongly to these two isolates (Figure 1), and C3b and C4b deposition was only slightly affected (Figure 4), while the sensitivity to the bactericidal activity was strongly reduced (Figure 3).

## Discussion

Our results show that natural populations of *N. meningitidis* are remarkably diverse during rapid spread of clones in the course of epidemics. The capsule synthesis locus has been found to be a hot spot of point mutations [12], and here we have analyzed the phenotype of 13 isolates with non-synonymous SNPs or stop codons in the capsule synthesis locus identified among 100 closely related A:ST-7 and A:ST-2859 isolates. Lack of O-acetylation in one *csaC* mutant and lack of capsule expression in four *csaB, csaD* and *ctrE* mutants abrogated binding and bactericidal activity of capsule specific antibodies. In two isolates with non-synonymous point mutations in the *csaB* gene encoding the capsule polymerase, the capsule expression was increased while the bactericidal activity of both capsule- and LPS-specific antibodies was strongly reduced. Absence of mutants with synonymous point mutation in the capsule locus in the analyzed bacterial populations [12] is speaking for (immune)selection of these capsule mutants.

While there is no doubt that the lack of capsule production in isolate 1921 is related to the stop codon in the *csaB* gene encoding the capsule polymerase, we have no formal proof that the non-synonymous mutations in *csaB* did directly result in lack of capsule expression in isolate 2206, reduced expression in isolates 2856 and 2857 or increased expression in strains 2025 and 2008. Strikingly however, the two over-producer isolates shared the same amino acid exchange (I82V) and also the two isolates with reduced capsule production shared the same (P143S) exchange. Future analysis of the structure of the polymerase and analyzes with recombinantly expressed mutant versions of the enzyme carrying the observed amino acid exchanges could provide molecular insight into structure-activity relation of the polymerase.

Polysaccharide capsules are thought to protect meningococci from phagocytic killing, opsonisation and complement-mediated bactericidal activity [29]. They are therefore regarded as a key virulence factor of invasive strains and represent the target of the currently available vaccines against serogroup A, C, Y and W meningococcal invasive disease [30]. After the introduction of the meningococcal serogroup A polysaccharide vaccine MenAfriVac [31–33], W, C, X and Y clones represent the main etiological agent of epidemics in the meningitis belt [34]. Therefore, it is important to understand the dynamics of the capsule synthesis genes and how mutations in this locus translate into phenotypic, structural and functional effects. Changes in capsule expression may in particular affect the bactericidal activity of capsule specific antibodies, which represent a correlate of protection for the capsule polysaccharide based vaccines.

In industrialized countries, the carried meningococcal populations are complex, temporarily relatively stable in composition and are comprising up to 50% serologically nongroupable isolates [35]. These include lineages harboring the capsule null locus lacking the genes required for capsule synthesis and transport [36], which are rarely associated with invasive disease [37]. In the African meningitis belt, lack of a diverse and temporally stable resident meningococcal pharyngeal flora is a characteristic feature of meningococcal colonization [14]. A small proportion of capsule deficient mutants related to the temporally dominating capsule-expressing isolates are commonly found [15,38]. They may be able to persist longer in the nasopharynx when a carrier has developed a capsule specific secretory antibody response. Furthermore, absence of capsule has been shown to generally facilitate the intimate attachment of meningococci to the human nasopharyngeal epithelium [30,39] via adhesins such as Opa and Opc [40], which are more efficiently shielded by the capsule than pili [41]. Among the 11 carriage mutants with point mutations in the capsule locus analyzed here, we found four isolates (1976, 2389, 1921, and 2206) with mutations in either the *ctrE, csaB* or *csaD* genes that lead to lack of capsule polysaccharide expression. Another four carriage isolates (2856, 2857, 2717, and 2617) with mutations in *csaA* or *csaB* genes produced reduced amounts of capsule. Thus, the serogroup A carriage population comprised a high proportion (8%) of mutants with complete or partial loss of capsule production, which may allow them to adhere more efficiently to the nasopharyngeal epithelium [30,39].

One of the two case isolates with mutations in capsule synthesis genes (2008), and also one carriage mutant (2025) showed an approximately 1.5-fold increase in capsule production compared to the reference isolates. These two capsule overproducing isolates with identical non-synonymous mutations in the *csaB* gene encoding a capsule polymerase linking ManNAc-phosphate monomers showed enhanced resistance to the bactericidal activity of both anti-LPS and anti-capsular antibodies. The increased amount of polysaccharide may reduce the bactericidal activity of the terminal complex of complement by enhanced shielding of the cell membranes [29] and interfere with antibody recognition of deeper cell surface structures, such as LPS. Increased amounts of capsule have also been shown to physically interfere with the engagement of C1q with the Fc portions of bound antibodies [42]. Similarly, hyperencapsulated C:ST-11 isolates from Spain have been found to be resistant to the bactericidal activity of vaccine induced anti-capsule antibodies [43]. In these strains insertion of an IS element has been associated with the transcriptional upregulation of both the *css* and the *ctr* operons.

The serogroup A capsular polysaccharide is a homopolymer of *N*-acetyl-mannosamine-phosphate-linked α1-6. O-acetylation is an important final step modification of the A polysaccharide polymer, and a dramatic reduction in immunogenicity is associated with the removal of the O-acetyl groups [19,44]. Many pathogenic bacteria are O-acetylated, but CsaC has no homology to other known O-acetyltransferases or to the sialic acid serogroup C, Y, and W capsule *O*-acetyltransferases (OatC and OatWY) [17]. In our study, we found that the carriage isolate 1666 had a stop mutation in the *csaC* gene coding for the meningococcal serogroup A O-3 and O-4 ManNAc acetyltransferase [17]. Neither the anti-A capsule mAb nor the polyclonal anti-capsular antiserum elicited by the ACWY conjugate vaccine Menveo recognized the non-O-acetylated polysaccharide resulting in a complete lack of bactericidal activity of the anti-ACWY capsule antiserum. In line with this, it has been observed [44] that the majority of the antibodies elicited by vaccination with a conjugate serogroup A polysaccharide vaccine were specific for epitopes involving O-acetyl groups. In contrast, the O-acetyl groups on the meningococcal serogroup C polysaccharide are not necessary for induction of protective antibodies [45]. Our results, therefore, underline the importance of O-acetylation for the immunogenicity of the *N. meningitidis* serogroup A polysaccharide. Loss of O-acetylation may thus represent an immune evasion mechanism.

Taken together, our results demonstrate that variation in the amount and structure of the expressed capsular polysaccharide due to point mutations is common in hypervirulent serogroup A meningococcal populations. In general, the genomic diversity of epidemic *N. meningitidis* lineages is extensive and is primarily attributed to the natural competence of the meningococci for transformation and homologous recombination rather than point mutation [12]. A reversion of the capsule mutants to encapsulated and O-acetylated phenotypes may therefore potentially occur rather by horizontal gene transfer than by reverse mutation. Reduction or loss of capsule polysaccharide production may bring advantages with respect to colonization efficacy, while capsule overproduction or lack of O-acetylation may increase resistance against host immune defense mechanisms during invasion of the bloodstream. Further studies are required to determine the impact of diversity in capsule expression on transmission dynamics and colonization patterns. Variation of capsule expression is of importance both for vaccine induced anti-capsular immunity and for meningococcal diagnostics, speaking for a combination of serogrouping and genotyping diagnostic approaches.

## Abbreviations

BA: Bolgatanga District
DPBS-BSA: Dulbecco’s phosphate buffered saline – bovine serum albumin
CFU: colony-forming units
cps: capsule synthesis locus
ELISA: enzyme-linked immunosorbent assay
HPAEC-PAD: high-performance anion-exchange chromatography with pulsed amperometric detection
HR-MAS NMR: high-resolution magic angle spinning nuclear magnetic resonance
KND: Kassena-Nankana District
LPS: lipopolysaccharide
mAb: monoclonal antibody
MH: Mueller-Hinton
MLST: multilocus sequence typing
NHD: Nouna _Health District
SNPs: single-nucleotide polymorphism
ST: sequence type

## Trademark statement

Menveo is a trademark owned by or licensed to the GSK group of companies.

MenAfriVac is a trademark of Serum Institute of India Private Ltd.

## Supplementary material

Supplemenatary data for this article can be accessed here.
