# Supplementary material for: Spontaneous point mutations in the capsule synthesis locus leading to structural and functional changes of the capsule in serogroup A meningococcal populations
Source: Virulence. 2018 Aug 1;9(1):1138–49. doi: 10.1080/21505594.2018.1467710 (PMC6086313; doi:10.1080/21505594.2018.1467710)
Supplement: Supplemental Material [file kvir-09-01-1467710-s001.zip › Supplementary Figure 1 -Caption.docx]

**Supplementary Figure 1.** Flow cytometric analysis of capsule expression in five ST-7 and six ST-2859 isolates randomly selected from the set of 87 serogroup A ST-7 and ST-2859 meningococci with no mutations in the capsule synthesis locus [12]. Binding of anti-A capsule mAb (A) and anti-ACWY capsule antiserum (B) is shown. Gray filled histogram: isolate 1446 incubated only with the secondary antibody Alexa Fluor 488. Purple: 1550; blue green: 1797; orange: 1446; pink: 1910; gray: 1361; red: 2187; green: 2263; black: 2813; blue: 2701; brown: 2432 and dark green:2857.
