# Supplementary figures and images for: Spontaneous point mutations in the capsule synthesis locus leading to structural and functional changes of the capsule in serogroup A meningococcal populations
Source: Virulence. 2018 Aug 1;9(1):1138–49. doi: 10.1080/21505594.2018.1467710 (PMC6086313; doi:10.1080/21505594.2018.1467710)

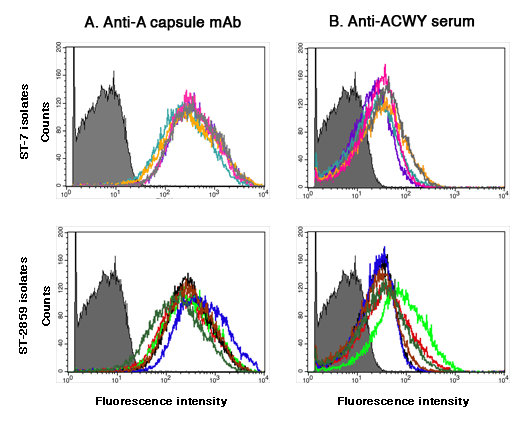

Supplement: Supplemental Material [file kvir-09-01-1467710-s001.zip › supplementary_figure_Ispasanie.tif]
